# Disseminated Intravascular Coagulation as an Initial Manifestation of Metastatic Prostate Cancer Emergently Treated with Docetaxel-Based Chemotherapy

**DOI:** 10.1155/2019/6092156

**Published:** 2019-04-24

**Authors:** Kavita Agrawal, Nirav Agrawal, Levin Miles

**Affiliations:** ^1^Department of Internal Medicine, Overlook Medical Center, 99 Beauvoir Avenue, Summit, NJ, USA; ^2^Department of Pathology, Overlook Medical Center, 99 Beauvoir Avenue, Summit, NJ, USA

## Abstract

A 70-year-old male presented with hematuria and bruising of arms and legs for the last three days. He also complained of urinary frequency and hesitancy and weight loss of 40 pounds over a span of four months. Initial blood tests showed prothrombin time (PT) of 25.1 seconds, international normalized ratio (INR) of 2.5, partial thromboplastin time (PTT) of 43.9 seconds, fibrinogen of 60 mg/dl, fibrin degradation products (FDP) of more than 20 *μ*g/ml, and platelets of 88,000/*μ*l. The impression was disseminated intravascular coagulation (DIC). A search was initiated to determine the underlying etiology precipitating DIC. Due to urinary symptoms and weight loss, prostate-specific antigen (PSA) was ordered. PSA was elevated at 942 *μ*g/dl. Computed tomography (CT) of the abdomen and pelvis without contrast showed an enlarged prostate with mass effect on the bladder base, left-sided hydronephrosis, and numerous enlarged pelvic lymph nodes. A bone scan of the whole body showed increased sclerosis of the L3 vertebral body. There was a concern for metastatic prostate cancer precipitating DIC. On first admission, our patient's DIC was stabilized with FFP and cryoprecipitate transfusions. He refused chemotherapy, and degarelix was not economically feasible. Accordingly, he was started on androgen deprivation therapy (ADT), bicalutamide, and leuprolide as an inpatient, pending the tissue biopsy. The patient refused a prostate biopsy. A bone marrow biopsy was performed which confirmed metastatic prostate adenocarcinoma. The patient was stable for discharge with a plan for outpatient chemotherapy. Subsequently, he was lost to follow-up with the oncology. Six months after the initial presentation, he was readmitted with hematuria. Repeat PSA worsened to 1,970 *μ*g/dl. Blood work was consistent with acute DIC. He refused chemotherapy again. So, he was restarted on ADT. However, his hematuria and DIC panel were worsening. He was emergently started on docetaxel as an inpatient (after patient agreement). Within three days of starting chemotherapy, his hematuria resolved and DIC panel showed consistent improvement.

## 1. Introduction

DIC is the most common coagulopathy manifested in prostate cancer. The estimated incidence is between 13 and 30% [1]. However, the clinical signs of DIC are actually found in only 0.4-1.65% of patients with prostate cancer [1].

Our report highlights a rare initial presentation of prostate cancer as DIC. It emphasizes the importance of monitoring the patients with metastatic prostate cancer for signs and symptoms of development of DIC as it can be life-threatening. Also, it shows successful use of docetaxel as an emergent treatment option to control DIC in metastatic prostate cancer.

## 2. Case Report

A 70-year-old male presented with hematuria and bruising of arms and legs for the last three days. He complained of unintentional weight loss of 40 pounds over the last four months. He also noted to have urinary frequency and hesitancy for four months. He denied nocturia, urinary dribbling, dysuria, or sensation of incomplete emptying of the bladder. He denied fever, chills, nausea, vomiting, abdominal pain, bowel complaints, or prior history of bleeding. He denied use of any blood thinners or nonsteroidal anti-inflammatory medications (NSAIDS).

He had past medical history of diabetes mellitus type 2 complicated by erectile dysfunction and hyperlipidemia. He had past surgical history of abdominal hernia repair. He denied smoking, alcohol, or recreational drug use. His medications included glipizide, metformin, tadafil, and atorvastatin. He denied family history of bleeding disorders or cancer.

Physical examination revealed an obese male patient in no acute distress. His vitals were within normal limits. Oral mucosa was moist. No lymphadenopathy was noted on examination. Lungs were clear to auscultation bilaterally. Heart sounds, rate, and rhythm were regular. The abdomen was soft, nontender, and nondistended with no hepatosplenomegaly. Cranial nerves 2-12 were grossly intact. Large ecchymoses measuring 3 × 3 cm on the anterior aspect of the right arm and 7 × 5 cm on the posterior aspect of the right lower leg were present. No rash or joint swelling was noted.

On admission, complete blood count (CBC) revealed a hemoglobin (Hb) level of 8.4 g/dl, white blood cell (WBC) count of 8,170/nl, and platelet count of 88 × 10^3^/*μ*l. The peripheral smear showed moderate red cell anisocytosis with few teardrop cells and rare schistocytes. Few giant platelets were noted. WBC were morphologically normal. Further workup showed PT of 25.1 seconds, INR of 2.5, APTT of 43.9 seconds, fibrinogen of 60 mg/dl, and FDP of more than 20 *μ*g/ml (Table 1). The impression was that the patient was in DIC. On admission, he was transfused FFP and cryoglobulin. On day 2 after initial presentation, his INR improved to 1.60 and fibrinogen to 114 mg/dl which remained stabilized on subsequent days without further transfusions (Table 1).

A search was initiated to determine an underlying etiology precipitating DIC. Based on the history of urinary complaints and unintentional weight loss, PSA was ordered. His PSA was elevated at 942 *μ*g/dl. CT of the abdomen and pelvis without contrast showed a significantly enlarged prostate with mass effect on the bladder base, mild left side hydroureteronephrosis to the level of the left ureterovesical junction, and numerous enlarged pelvic lymph nodes. A bone scan of the whole body showed increased sclerosis of the L3 vertebral body suggesting osseous lesions. Based on the above findings, there was a concern for metastatic prostate cancer driving DIC.

**Figure 1:**
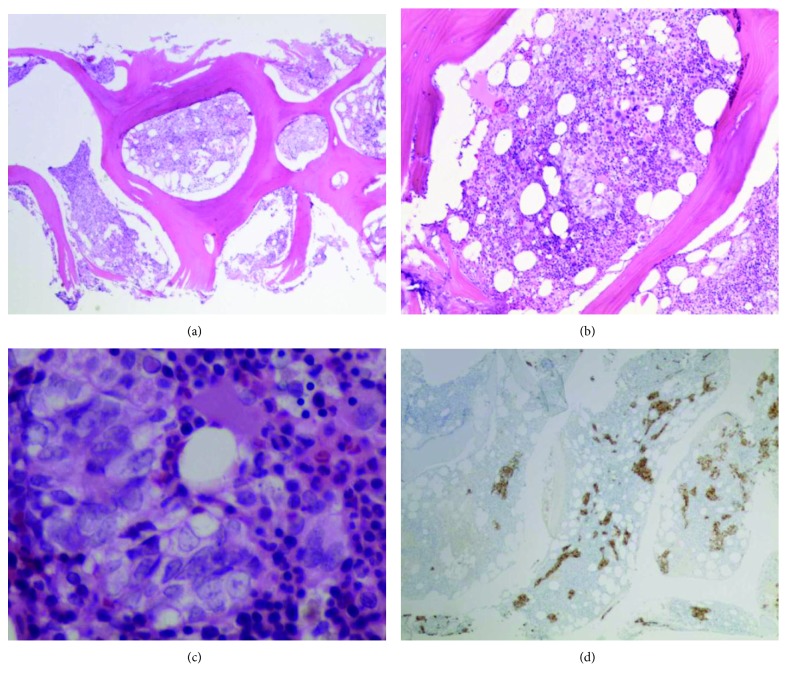
(a) Low-power bone marrow (H&E, ×40) showing clusters of epithelioid cells on small aggregates within hypercellular bone marrow. (b) High-power bone marrow (H&E, ×200) showing clusters of epithelioid cells on small aggregates within hypercellular bone marrow. (c) High-power bone marrow (H&E, ×600) showing clusters of epithelioid cells on small aggregates within hypercellular bone marrow. (d) Low-power bone marrow with OSCAR stain highlighting metastasis.

On day 5 after the initial presentation, the patient was started on ADT consisting of bicalutamide 50 mg daily and leuprolide 7.5 mg subcutaneously on monthly basis as an inpatient. At this time, the tissue biopsy was pending. He refused a prostate biopsy. Upon further discussion, he agreed for a bone marrow biopsy. It showed a hypercellular marrow due to metastatic infiltrate comprising 40% of marrow cellularity (Figures 1(a)–1(d)). Residual hematopoiesis was present but decreased. Islands of erythroid precursors appeared adequate. Megakaryocytes showed some clustering with increased atypical forms. Immunohistochemical stains showed neoplastic cells dimly positive for PSA and prostate-specific membrane antigen (PSMA). The findings were consistent with metastatic prostate adenocarcinoma. By day 6 of the initial presentation, hematuria was resolved. He had no other signs of active bleeding. His hemoglobin was stable at around 8 g/dl. DIC labs were stable (Table 1). He was thus discharged from the hospital on ADT. The plan was to closely follow-up with his oncologist and to start chemotherapy as an outpatient.

On day 11 after the initial presentation, the patient was seen in the oncologist's office. He denied any bleeding episodes or new bruising. Lab work showed PT of 15 seconds, INR of 1.1, APTT of 26.6 seconds, fibrinogen of 151 mg/dl, and platelets of 161 × 10^3^/*μ*l. The oncologist had an extensive discussion with the patient about the risks and benefits to start chemotherapy with docetaxel along with ADT. But the patient was hesitant to undergo chemotherapy. So, he was continued on ADT only. He was instructed to follow-up in one month with a plan to recheck PSA and DIC labs at that time. Subsequently, he was then lost to follow-up with the oncologist.

Six months after the initial presentation, he was readmitted to our hospital with hematuria for one week. He denied bruising or bleeding from any other sites. On readmission, blood work showed PT of 22.5 seconds, INR of 2.0, APTT of 52.6 seconds, fibrinogen of 98 mg/dl, and FDP of more than 20 *μ*g/ml (Table 1). CBC showed Hb of 8.0 g/dl and platelet count of 63 × 10^3^/*μ*l. PSA was repeated which was 1,970 *μ*g/dl. His testosterone level was 118 ng/dl. At this time, he was off ADT for 5 months. The impression was reactivation of DIC secondary to metastatic prostate cancer with medication noncompliance.

During this time, the oncologist had repeat discussion with the patient about the necessity to start chemotherapy, but he refused. So, he was restarted on ADT. However, his hematuria was worsening. During the hospital course, his Hb was gradually trending down with the lowest value of 6.3 g/dl. Also, on day 10 after second readmission, his DIC labs started worsening (Table 1). He was supported with multiple packed red cells, platelets, FFP, and cryoprecipitate transfusions. Due to concern of worsening DIC with ADT, the plan was to start inpatient chemotherapy with docetaxel for more cytoreduction of prostate cancer (patient agreed after rediscussion). On day 12 after second readmission, he was started on docetaxel 60 mg/m^2^ every 3 weeks (typical dose of docetaxel is 75 mg/m^2^ which was reduced by 20% secondary to thrombocytopenia). By day 15 after second readmission, his DIC panel showed consistent improvement except persistent thrombocytopenia. Hematuria was resolved. Thrombocytopenia was attributed to chemotherapy-related side effect. Accordingly, the dose of docetaxel was adjusted. He was deemed stable for discharge with close outpatient follow-up with oncology.

As of the writing of this case, the patient has completed 2 cycles of docetaxel with ADT. After 2 cycles of chemotherapy, repeat PSA trended down to 1,070 *μ*g/dl. The testosterone level was 12.5 ng/dl. DIC panel was within normal limits.

## 3. Discussion

DIC is a systemic condition characterized by widespread activation of clotting factors which results in intravascular formation of fibrin. This causes thrombotic occlusion of blood vessels which compromises blood supply to various organs and contributes to multiorgan dysfunction. At the same time, consumption of coagulation factors and platelets can cause severe life-threatening bleeding [2]. DIC does not occur in isolation. It is crucial to search for underlying causes propagating DIC. Sepsis is the most common cause of DIC. Obstetrical complications such as abruptio placenta, preeclampsia, hemolysis, elevated liver enzymes, and low platelet count (HELLP syndrome), and amniotic fluid embolism can also lead to DIC. Malignancies such as acute promyelocytic leukemia, brain tumor, and mucinous tumors (ovarian, pancreatic, and gastric) are more commonly associated with DIC. Other causes of DIC include blood transfusion reactions, trauma, surgery, solid organ transplant rejection, snake bites, and vascular abnormalities such as aortic aneurysm [3].

DIC is the most common coagulation complication observed in prostate cancer patients. The incidence of DIC in prostate cancer is reported to be between 13 and 30% [1]. However, the clinical signs of DIC are apparent in only 0.4-1.65% of patients with prostate cancer [1]. Other coagulopathies associated with prostate cancer include thrombotic thrombocytopenic purpura, thrombosis, Trousseau's syndrome, and acquired factor VIII inhibitor development.

The pathophysiology of DIC in prostate cancer is not well understood. Several studies have postulated that cancer cells express procoagulant molecules such as tissue factor (TF) on the surface membrane. TF forms a complex with factor VII which activates coagulation cascade causing consumption of coagulation proteins and leading to DIC. This hypothesis was further strengthened by identifying the microparticles containing TF in the blood samples of patients with malignancy [4]. Cancer cells have also been shown to express molecules called cancer procoagulant (CP). CP is a calcium-dependent cysteine protease that directly activates factor X leading to DIC. Nickel et al. have illustrated a critical role of polyphosphate/factor XII- triggered coagulation cascade in prostate cancer patients culminating into DIC [5]. Prostate cancer cells secrete prostasomes (small vesicles) expressing long chains of polyphosphates on the surface membrane. These polyphosphates form a complex with factor XII and activate a coagulation cascade.

Most patients with DIC secondary to prostate cancer are clinically asymptomatic. They are in chronic DIC which is apparent on laboratory testing. The clinical symptoms of DIC are present in only a small percentage of patients with prostate cancer as mentioned above. Clinically, patients may be felt to have bleeding predominant or thrombotic predominant DIC based on where complications are manifested. The bleeding manifestations include ecchymosis, petechiae, blood oozing from mucosal surfaces or wound sites, or perioperative bleeding. It can also present as life-threatening bleeding such as gastrointestinal or intracranial bleeding. The thrombotic manifestations include venous or arterial thromboembolism complicated by organ ischemia. The laboratory abnormalities in chronic DIC include normal or mildly elevated PT, APTT, and fibrinogen levels. Platelet counts may be normal or mildly reduced. D-dimer and FDPs may be elevated. In acute DIC, patients will have prolonged PT and APTT, reduced fibrinogen, thrombocytopenia, and elevated D-dimer and FDPs.

The mainstay of the management of DIC is the treatment of underlying cause which is prostate cancer in our case. It is crucial to control the etiology which is driving DIC. The management also involves supportive treatment based on the bleeding predominant or thrombosis predominant state of DIC.

Platelet transfusion is warranted in patients with active bleeding or at high risk of bleeding such as after surgery or those requiring invasive procedures when the platelet count is less than 50,000/*μ*l [6]. In patients with no bleeding or not requiring invasive procedures, platelet transfusion should be given only when platelet count is less than 10,000-20,000/*μ*l [6]. This is because these patients are at increased risk for spontaneous bleeding.

Also, in patients who are actively bleeding or need to undergo invasive procedure and have prolonged PT/APTT (>1.5 times normal) or fibrinogen less than 100 mg/dl should be given FFP or cryoprecipitate transfusions, respectively [6]. Prothrombin complex concentrate (PCC) can be considered in situations with concern for fluid overload due to FFP transfusions. However, it is important to remember that PCC contains only four coagulation factors (factors II, VII, IX, and X). So, it will only partially treat the coagulation abnormalities in DIC. Except for where fluid overload is a concern, FFP should be used instead of PCC as the latter can lead to higher thrombotic complications when compared to treatment with FFP [7, 8]. Vitamin K can be administered to replenish vitamin K-dependent factors such as factors II, VII, IX, and X and proteins C and S.

Antifibrinolytic agents such as tranexamic acid or epsilon aminocaproic acid (EACA) can be considered in patients with severe bleeding due to a hyperfibrinolytic state [6]. The hyperfibrinolytic state can be indicated by high levels of alpha-2 antiplasmin complex levels.

In DIC patients with predominant thrombosis, studies have shown benefit with therapeutic doses of heparin infusion. In critically ill, nonbleeding DIC patients, it is recommended to give prophylactic doses of unfractionated heparin or low molecular weight heparin for venous thromboembolism prophylaxis [6].

The therapeutic options that are reported to be effective in treating patients with DIC secondary to prostate cancer include hormonal therapy, radiopharmaceutical therapy, and chemotherapy. The most widely used hormonal approach is treatment with gonadotropin-releasing hormone (GnRH) agonists such as leuprolide and goserelin. The initial use of GnRH agonists causes a transient surge of luteinizing hormone (LH) before suppressing LH levels. This surge can stimulate testicular testosterone production causing worsening of the disease. This is called a flare phenomenon. GnRH agonists combined with antiandrogens such as flutamide and bicalutamide can prevent the flare phenomenon.

Degarelix is a new rapidly acting GnRH antagonists that has shown benefit in management of metastatic prostate cancer [9]. Degarelix directly binds to pituitary gonadotropin-releasing cells and suppresses LH and thereby testosterone without causing the flare phenomenon. So, it does not require combination with antiandrogen therapy. Studies have shown that the immediate onset of action of degarelix causes more rapid suppression of LH and testosterone levels compared to leuprolide [9].

Hormonal therapies that have shown to be effective in controlling DIC related to castration-resistant prostate cancer include ketoconazole [10, 11], abiteratone, and estrogen analogues such as diethylstilbestrol. Ketoconazole, an antifungal agent, is a nonselective inhibitor of 17-alpha hydroxylase which inhibits adrenal synthesis of testosterone. Ketoconazole is a rapidly acting agent and has been shown to reach castration levels in 24 hours. Abiraterone is a selective and irreversible inhibitor of 17-alpha hydroxylase which inhibits both adrenal and testes testosterone productions. A clinical trial has shown a survival benefit of abiraterone plus prednisone in men who had previously been treated with docetaxel and in those who are chemotherapy naive [12, 13].

Radiopharmaceutical therapy such as Samarium 153 has been reported to be successfully used in emergency treatment of DIC in metastatic castration-resistant prostate cancer [1].

Chemotherapeutic agents can also be considered to control DIC in metastatic prostate cancer. Upon our literature review, we came across few reports. Smith reported 2 patients successfully treated with mitoxantrone-containing chemotherapy [14]. Avances et al. reported a case treated with combination of docetaxel and cisplatin [15]. We found 3 reports of patients with metastatic prostate cancer presenting as DIC with thrombocytopenia and bleeding who were successfully treated with docetaxel-based chemotherapy [16–18].

Our patient presented with DIC as the initial manifestation of metastatic prostate cancer. On first admission, our patient's DIC was stabilized with FFP and cryoprecipitate transfusions. To prevent future clinical exacerbations of DIC, degarelix would have been our agent of choice. This is because degarelix does not cause a flare phenomenon and has an immediate onset of action. However, it was not economically feasible in our patient. Accordingly, on day 5 after initial presentation, he was started on bicalutamide and leuprolide. He was discharged the next day with a close outpatient follow-up. Subsequently, he was lost to follow-up. Six months later, he was readmitted with similar presentation as his first admission. He refused chemotherapy, so he was restarted on bicalutamide and leuprolide. However, his hematuria and DIC panel were worsening. He was emergently started on docetaxel-based chemotherapy as an inpatient (after patient agreement). Three days after starting chemotherapy, he had resolution of DIC and his hematuria resolved. The patient continued to have low platelet count which was attributed to side effect from chemotherapy. Accordingly, the dose of chemotherapy was adjusted.

## 4. Conclusion

Our report illustrates a case of DIC as an initial manifestation of metastatic prostate cancer. This is a rare presentation which accounts for 0.4-1.65% of prostate cancer patients. It is important to monitor the patients with metastatic prostate cancer for signs and symptoms of development of DIC such as bleeding or low platelet counts because it can be life-threatening. This case also adds to our limited literature on the treatment strategies of metastatic prostate cancer-induced DIC. We believe that docetaxel-based chemotherapy can be successfully used as an emergent treatment of DIC in metastatic prostate cancer patients with bleeding and thrombocytopenia.

## Figures and Tables

**Table 1a:** (a) Trend of laboratory abnormalities on 1^st^ admission

	PT/INR (seconds)	PTT (seconds)	Fibrinogen (mg/dl)	FDP (*μ*g/ml)	Platelet (per *μ*l)	Event
Reference range	12.0-15.0	23.0-37.0	227-467	<5	150-450	
Day 1	25.1/2.5	43.9	60	>20	88,000	Hematuria × 3 days; supportive transfusions
Day 2	18.8/1.60	39	114	>20	89,000	
Day 3	18.4/1.56	36.9	119	>20	99,000	
Day 5	18.5/1.57	35.5	104	>20	104,000	ADT started
Day 6	16.0/1.29	28.8	149	>20	104,000	Discharged; advised outpatient follow-up

**Table 1b:** (b) Trend of laboratory abnormalities on 2^nd^ admission

Day 1	22.5/2.03	52.6	98	>20	63,000	Hematuria × 1 week; restarted ADT
Day 5	25.9/2.45	52.9	147	>20	51,000	Supportive transfusions
Day 10	22.1/1.98	51.6	81	>20	47,000	Hematuria worsening; supportive transfusions
Day 11	23.5/2.15	45.5	60	>20	41,000	
Day 12	30.4/3.03	48.4	60	>20	36,000	Chemotherapy started
Day 13	23.5/2.15	45.4	69	>20	48,000	
Day 14	20.0/1.74	56.8	133	>20	35,000	
Day 15	20.4/1.78	53.1	152	>20	30,000	
